# Adaptive vibration control method for double-crystal monochromator base on VMD and FxNLMS

**DOI:** 10.1107/S1600577523000528

**Published:** 2023-02-20

**Authors:** Yang Bai, Xuepeng Gong, Qipeng Lu, Yuan Song, Wanqian Zhu, Song Xue, Dazhuang Wang, Zhongqi Peng, Zhen Zhang

**Affiliations:** a Changchun Institute of Optics, Fine Mechanics and Physics, Chinese Academy of Sciences, Changchun 130033, People’s Republic of China; b University of Chinese Academy of Sciences, Beijing 100049, People’s Republic of China; c Shanghai Advanced Research Institute, Chinese Academy of Sciences, Shanghai 201204, People’s Republic of China; Bhabha Atomic Research Centre, India

**Keywords:** active vibration control, double-crystal monochromator, FxNLMS algorithm, variational modal decomposition, synchrotron radiation

## Abstract

Double-crystal monochromators (DCMs) are one of the most critical optical devices in beamlines at synchrotron sources, directly affecting the quality of the beam energy and position. As the performance of synchrotron light sources continues to improve, higher demands are placed on the stability of DCMs. This paper proposes a novel adaptive vibration control method combining variational modal decomposition (VMD) and filter-x normalized least mean squares (FxNLMS), ensuring DCM stability under random engineering disturbance. Firstly, the sample entropy of the vibration signal is selected as the fitness function, and the number of modal components *k* and the penalty factor α are optimized by a genetic algorithm. Subsequently, the vibration signal is decomposed into band frequencies that do not overlap with each other. Eventually, each band signal is individually governed by the FxNLMS controller. Numerical results have demonstrated that the proposed adaptive vibration control method has high convergence accuracy and excellent vibration suppression performance. Furthermore, the effectiveness of the vibration control method has been verified with actual measured vibration signals of the DCM.

## Abstract 2

With the performance of synchrotron radiation sources increasing significantly, higher requirements have been placed on the stability of double-crystal monochromators (DCMs). Using traditional passive vibration control techniques, such as optimized structures, elastic damping and vibration-absorbing materials, is unlikely to meet the future requirements of DCMs. The proposed method is the first attempt of an adaptive filtering algorithm based on variational modal decomposition in the field of DCMs development, which is an advancement for the development of high-performance DCMs at synchrotron radiation facilities.

## 1. Introduction

Nowadays, the large scientific research platform based on synchrotron radiation provides advanced experimental technology tools for many disciplines, such as life science, physics, chemistry, biology, medicine, materials, archeology, *etc*. (Jin *et al.*, 2017 ▸). Double-crystal monochromators (DCMs) are one of the key optical instruments on hard X-ray beamlines at synchrotron radiation facilities, separating the hard X-rays from the synchrotron radiation source. As the performance of synchrotron radiation sources significantly increases, higher requirements are placed on the stability of DCMs. Using traditional passive vibration control techniques, such as optimized structures, elastic damping and vibration-absorbing materials, is unlikely to meet the future requirements of DCMs (Yamazaki *et al.*, 2013 ▸; Baker *et al.*, 2013 ▸; Wu *et al.*, 2021*a* ▸,*b* ▸). Therefore, it is necessary to design and implement efficient active vibration control techniques to further improve the stability performance of DCMs.

**Figure 1:**
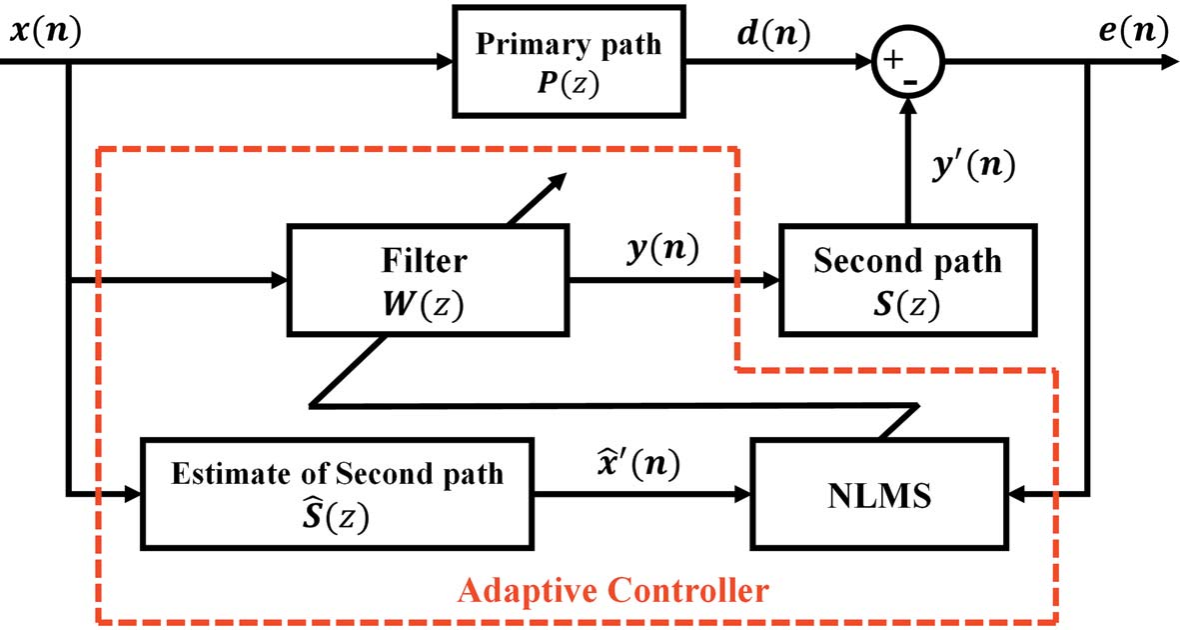
Block diagram of the FxNLMS algorithm.

The least-mean-square (LMS) algorithm is widely used in active vibration control (Sun *et al.*, 2007 ▸), and requires no precise mathematical model of the controlled object and can adjust the filter parameters adaptively based on system input and output response; it also has a simple structure and can be easily implemented, and has strong approximation capability for linear systems. However, when the input vector is relatively large, the LMS algorithm encounters the problem of noise amplification. The normalized LMS (NLMS) algorithm conquers this problem by normalizing the adaptive step size. The filter-x normalized least-mean-square (FxNLMS) algorithm based on adaptive filtering technology has been applied in the field of active vibration control due to its excellent convergence accuracy, powerful adaptive capability and robustness (Fallah & Moetakef-Imani, 2019 ▸; Yi *et al.*, 2019 ▸). The classical FxNLMS algorithm is illustrated in Fig. 1 ▸ The computational equation of the FxNLMS algorithm is shown in Table 1 ▸.

Empirical mode decomposition (EMD) is flawed by end effects, modal aliasing and over-enveloping; the short-time Fourier transform (STFT) processing signal cannot obtain high time-frequency resolution in the meantime; the local mean decomposition (LMD) also yields a false product function component (Wu & Huang, 2009) ▸; Bao *et al.*, 2020 ▸). To solve the above problem, the variational modal decomposition (VMD) algorithm was proposed by Dragomiretskiy & Zosso (2014 ▸). VMD screens the intrinsic mode function (IMF) components in the form of solving the optimal solution of the variational problem (Dragomiretskiy & Zosso, 2014 ▸). This method continuously iterates to search for the most suitable solution and adaptively achieves effective signal decomposition. Constructing and solving the constrained variational model to decompose the signal involves techniques such as the Wiener filter, Hilbert transform and frequency mixing. It is applied in mechanical fault diagnosis since it adopts a non-recursive conceptual framework. For instance, Wang *et al.* (2015 ▸) proposed a method to analyze faults by friction factor and applied variational model decomposition analysis, which is known to be more effective in diagnosis by comparison. Dey *et al.* (2015 ▸) combined VMD and Teager energy operators for fault diagnosis. The VMD algorithm makes the decomposition results stable by constructing the variational problem. However, VMD suffers from the deficiency that the decomposition effect is strongly influenced by the number of modal components *k* and the penalty factor α (Ram & Mohanty, 2017 ▸). When using the VMD method to process the vibration signal, the values of the number of modal components *k* and the penalty factor α are set empirically before the calculation. Therefore, the combination of [*k*, α] parameters of VMD is subject to artificial factors that can lead to over- or under-decomposition of the decomposition results. The value of the penalty factor α is important to ensure the accuracy of the VMD algorithm when reconstructing the signal. If the initial value of α is not set properly, the VMD algorithm will decompose the overlapping modal signals or the center frequency will be unstable, which may lead to incorrect decomposition of the algorithm and failure to obtain optimal resolution. Li *et al.* (2017 ▸) proposed an independent VMD method that found optimal modes by peak search and similarity principle. Wang *et al.* (2018 ▸) used the energy difference of the decomposed signal as a criterion to determine the preset modal parameters. The genetic algorithm (GA) is an optimization algorithm that simulates natural biological selection and genetic evolution. The algorithm consists of three genetic operators: selection, crossover and mutation. The GA can solve nonlinear problems quickly, efficiently and rapidly on a global scale (Singh & Harshit, 2014 ▸). Bian (2017 ▸) proposed a VMD method based on the GA to optimize the number of modal components *k* and the penalty factor α.

Based on the above introduction, this paper proposes a novel adaptive vibration control method based on VMD and FxNLMS to ensure the stability of ultra-precision optical instruments under random engineering disturbance. Since the VMD algorithm has a better de-correlation capability, VMD is implemented into the adaptive filtering algorithm. The extraction properties of VMD coefficients are used to decompose the input signal in the multi-scale space, reducing the dynamic spectral range of the adaptive filter’s auto-correlated array of input vectors. Consequently, the convergence speed and stability of the FxNLMS algorithm are improved. The primary process of this method is as follows: firstly, VMD is proposed to decompose the vibration signals into IMFs; then, the sample entropy of the vibration signal is selected as the fitness function, and the number of IMFs and penalty factor are optimized with GA; eventually, each IMF is controlled individually by the FxNLMS controller. Simulation results have demonstrated that the convergence accuracy and vibration suppression performance of the proposed method is much better than for the FxNLMS algorithm. Furthermore, the effectiveness of the method was verified with actual measured vibration signals.

## 2. The proposed algorithm

### 2.1. GA-VMD basic principle

The VMD process is essentially the solution process of the variational problem, which involves three critical concepts: classical Wiener filtering, Hilbert transform and frequency mixing (Dragomiretskiy & Zosso, 2014 ▸). The basic idea of the VMD algorithm is to search for a new way to adapt the signal decomposition process, which turns the decomposition process into a new constrained variational problem that can be decomposed. The VMD algorithm abandons the principle of refinement sieving of the modal signal in EMD. In this paper, the sample entropy is considered as the fitness function of the GA to optimize modal components *k* and the penalty factor α. The specific process is as follows.

For each modal function, the Hilbert transform is applied to the function to create a linear operator which can obtain the resolved spectral signal, expressed by

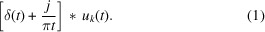

Mixing of all modal analysis signals is given by

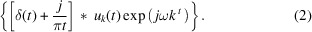

The constrained equation for the constructed variational model is given by (Dragomiretskiy & Zosso, 2014 ▸)

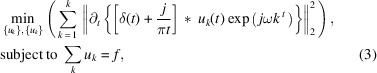

where δ(*t*) is the Dirichlet function; * is the convolution operation; {*u*_*k*_} = {*u*_1_,…, *u*_*k*_} is the set of IMFs obtained after the VMD decomposition of the modes; and {ω_*k*_} = {ω_1_,…, ω_*k*_} is the combination of the component center frequencies.

Introducing the penalty factor α and Lagrangian multiplier λ(*t*), which transforms the constrained variational problem into the unconstrained variational problem, the extended Lagrangian is expressed by

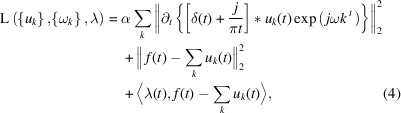

where α is the penalty factor and λ is the Lagrangian multiplier.

The variational problem is addressed by the alternate direction method of multipliers (ADMM) (Nocedal & Wright, 2006 ▸) and the optimal solution is obtained by updating 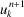 , 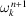 and λ^*n*+1^. 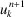 is denoted by

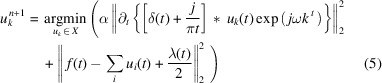

where *n* is the iteration.

The Parseval Fourier isometric transformation is utilized for equation (5), and the frequency domain range expression is given by

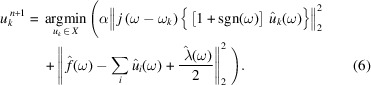

Equation (6) is converted into the form of the non-negative frequency interval integral, and then 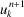 is given by

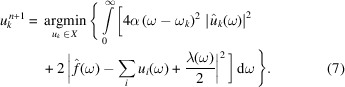

The optimal solution of the problem can be obtained as

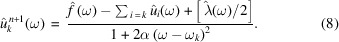

Base on a similar scheme, the central frequency problem is transformed to the frequency domain,

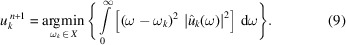

From equation (9), the updated formula of the center frequency is given by

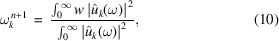

where 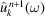 is equivalent to the Wiener filtering result of the current residual [ 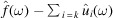 ].

The VMD algorithm is continuously updated in the frequency domain, after which the Fourier inversion is performed to obtain the results in the time domain. The practical procedure is illustrated as follows:

(1) Initialize 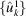 , 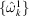 , 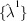 and *n* = 0.

(2) According to equations (8) and (10), update {*u*_*k*_}, {ω_*k*_}.

(3) Update the Lagrangian multiplier λ, which is given by

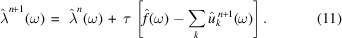




(4) The above iterations continue until convergence. The deterministic conditions is

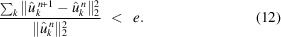

The sample entropy method is less dependent on data length and more resistant to interference, which has been widely applied in vibration signal research. The sample entropy of the vibration signal is selected as the fitness function of the GA, which is applied to determine whether the individual modal function components and penalty factors meet the decomposition requirements. The vibration signal *X*_*i*_(*n*) is a time series of length 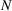 , *i* = 1, 2,…*N*. The vectors constructing *X*_*i*_(*n*) are 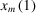 , 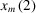 ,…, 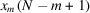 , given by

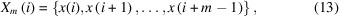

where *m* is the vector length.

The maximum value of the absolute value of the element-specific difference of two vectors is given by

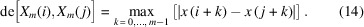

For individual *X*_*m*_(*i*), the difference between *X*_*m*_(*i*) and *X*_*m*_(*j*) is calculated to be less than that to the quantity *j* (1 ≤ *j* ≤ *N* − *m*, *j* ≠ *i*) of parameter *v*, defined as

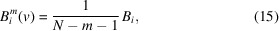




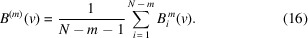

When the dimension is *m* + 1, the difference between *X*_*m*+1_(*i*) and *X*_*m*+1_(*j*) is calculated to be less than to the quantity *j* (1 ≤ *j* ≤ *N* − *m*, *j* ≠ *i*) of parameter *v*, defined as

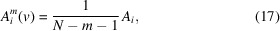




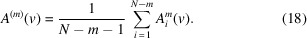


*B* ^(*m*)^(*v*) and *A* ^(*m*)^(*v*) are the *m* point probability and *m* + 1 point probability of being able to match the two sequences of elements under the similarity tolerance *v*. The sample entropy of this time series is defined as

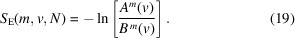

The dimension *m* is generally taken as *m* = 1–2; *v* = 0.1 std ≃ 0.25 std (where std is standard deviation of the data).

**Figure 2:**
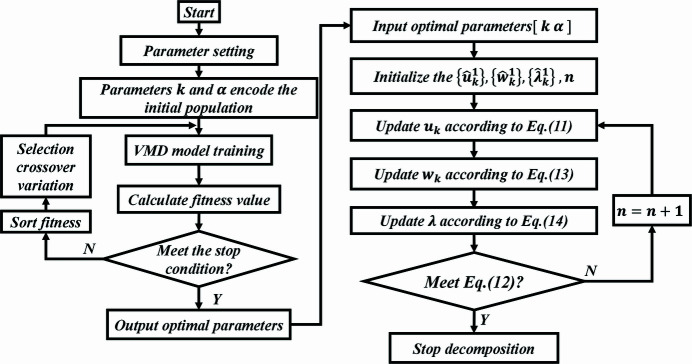
Schematic diagram of VMD parameter optimization by the GA.

The sample entropy is selected as the fitness function of the GA to adaptably obtain the [*k*, α] optimal parameter combination of the VMD, which realizes the adaptive determination of modal components *k* and the penalty factor α of the vibration signal for an ultra-precision optical instrument. A schematic diagram of VMD parameter optimization by GA is shown in Fig. 2 ▸.

### 2.2. Active vibration control algorithm

**Figure 3:**
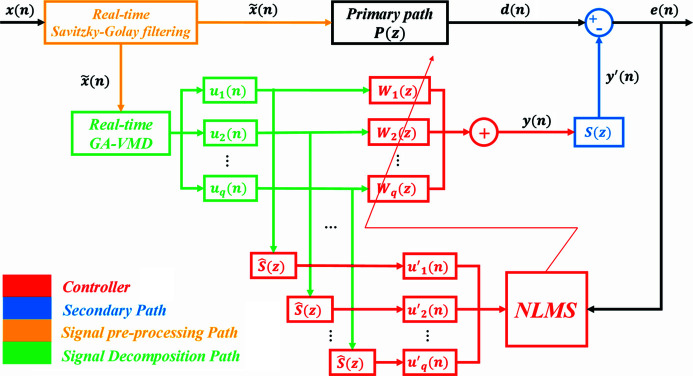
Schematic diagram of the adaptive vibration control method base on VMD and FxNLMS.

A schematic diagram of the adaptive vibration control method based on VMD and FxNLMS is shown in Fig. 3 ▸. Actual vibration signals are constantly subject to baseline drift (trend term of the signal) in the acquisition process caused by environmental interference. Since the correctness of the vibration signal analysis result is directly related to the trend term, it is necessary to pre-process the vibration signal to eliminate the tendency term. The Savitzky-Golay filter is a polynomial-based least-squares fitting filter method (Schafer, 2011 ▸), which has the advantage of following the variation of the baseline drift (Krishnan & Seelamantula, 2013 ▸). The specific process of adaptive vibration control method is as follows:

The vibration signal is 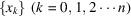 ; the function 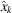 is set to be

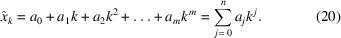

The square sum of *x*_*k*_ with 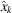 error is

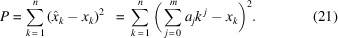

According to the extreme value condition of the least-squares method, the first-order partial derivative and second-order partial derivative of *P* with respect to *a*_*i*_ are 0, given by

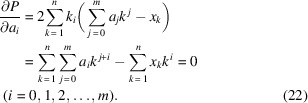

According to equation (22), *m* + 1 coefficients *a*_*j*_ can be established.

The signal 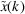 with the trend term removed is given by

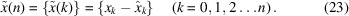

The input signal 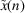 is decomposed by GA-VMD (VMD optimized by GA) into a series of signals with different frequency bands 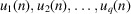 . As shown in Fig. 3 ▸, the output signal of the *i*th filter is given by

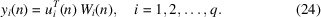

The error signal is given by

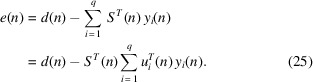

According to the Lagrangian optimality principle, the updated equation is given by

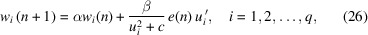

where α is the leakage factor (0 ≤ α ≤ 1); the leakage factor is introduced to restrict the power of the actuator to reduce nonlinear distortion. β is the convergence factor (0 ≤ β ≤ 2). In practice, the iteration step is large when 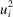 is excessively small. For double-precision floating-point inputs, *c* is 2.22044604925031341 × 10^−16^; for single-precision floating-point inputs, *c* is 1.192092896 × 10^−7^; for fixed-point input, *c* is 0 (Madisetti & Williams, 1999 ▸; Akhtar *et al.*, 2004 ▸).

### 2.3. Numerical simulation

In order to verify the effectiveness of the proposed adaptive vibration control method, numerical simulations were performed to demonstrate the accuracy of the method. The expression of the input simulation signal *x*(*t*) is

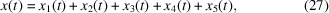

where

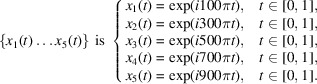




**Figure 4:**
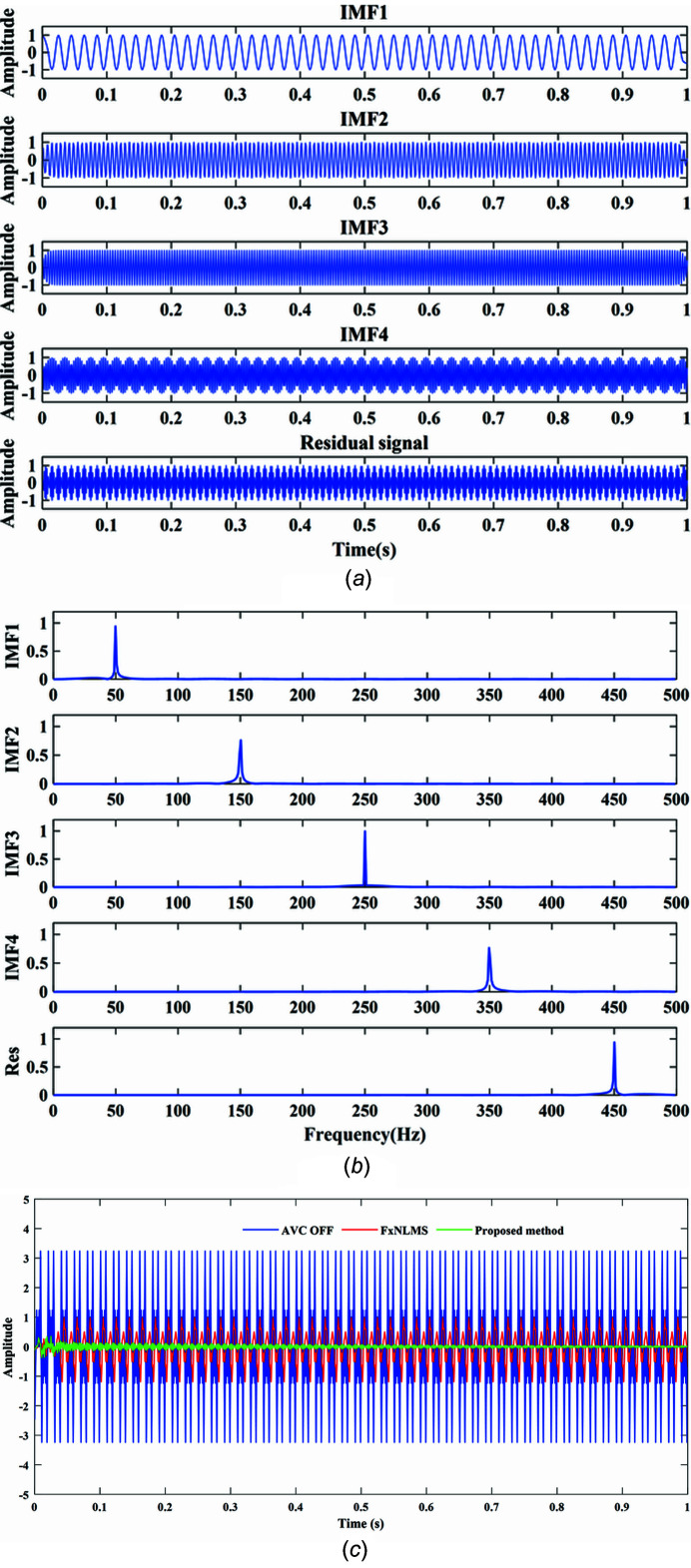
Numerical simulation results: (*a*) time domain IMFs; (*b*) frequency domain IMFs; (*c*) control results.

Fig. 4 ▸ shows comparison results of the vibration control under the harmonic superimposed signals. Figs. 4 ▸(*a*) and Fig. 4 ▸(*b*) show the input simulation signal decomposition IMFs time domain and frequency domain spectra. Three-fold harmonic, five-fold harmonic, seven-fold harmonic, nine-fold harmonic and fundamental harmonic components are carefully separated from mixed harmonic signal with peaks of 150 Hz, 250 Hz, 350 Hz, 450 Hz and 50 Hz. Obviously, the GA optimized VMD algorithm has been made more simplified and robust. Fig. 4 ▸(*c*) shows the convergence results of the FxNLMS algorithm within 1 second, which decreases the amplitude by 66.85%. Meanwhile, the proposed method vibration suppression performance achieves 99.95%.

## 3. Case validations

**Figure 5:**
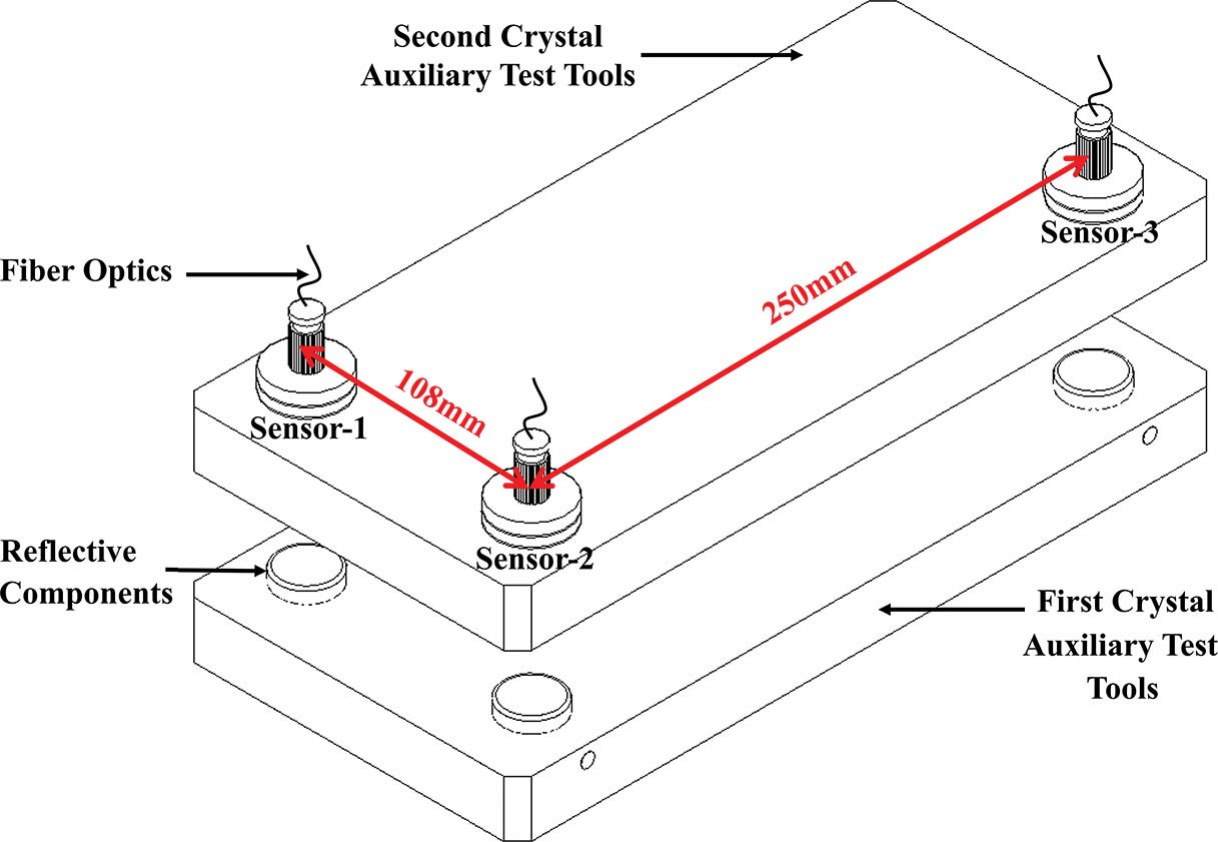
DCM field vibration measurement diagram.

To further validate the performance advantages of the proposed adaptive vibration control method over the FxNLMS algorithm, in addition to the simulated mixed signals in Section 2.3, experiments on the measured vibration signals generated by ultra-precise optical instruments (DCMs at synchrotron facilities) are reported in this section. A DCM field vibration measurement diagram is shown in Fig. 5 ▸; the measurement sensor parameters are shown in Table 2 ▸.

### 3.1. Case 1

**Figure 6:**
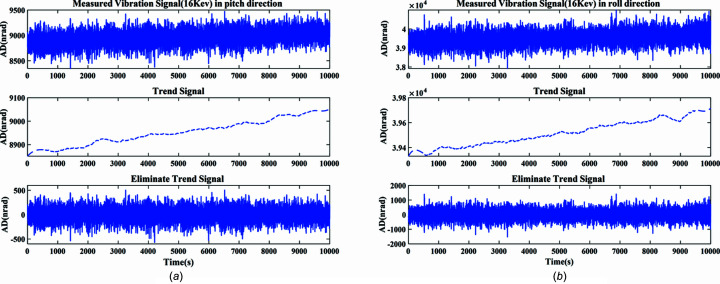
Actual measured signal of the DCM at Bragg@16 keV: (*a*) pitch direction; (*b*) roll direction.

**Figure 7:**
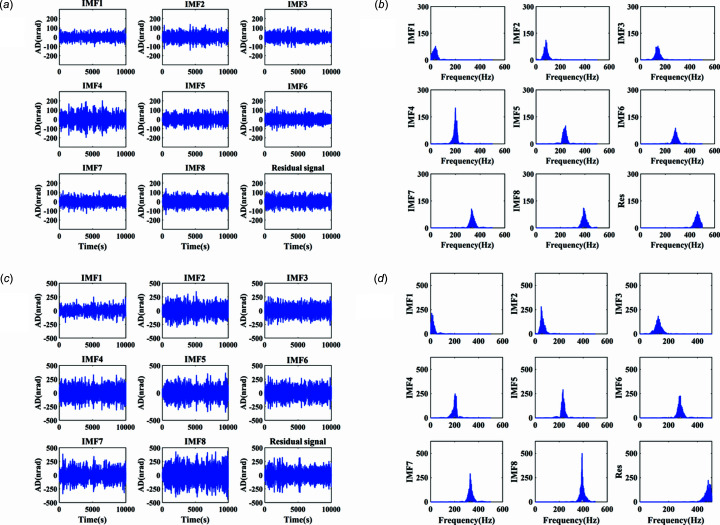
Decomposed signal and spectrum: (*a*) time-domain results in the pitch direction; (*b*) frequency-domain (0–500 Hz) results in the pitch direction; (*c*) time-domain results in the roll direction; (*d*) frequency-domain (0–500 Hz) results in the roll direction.

**Figure 8:**
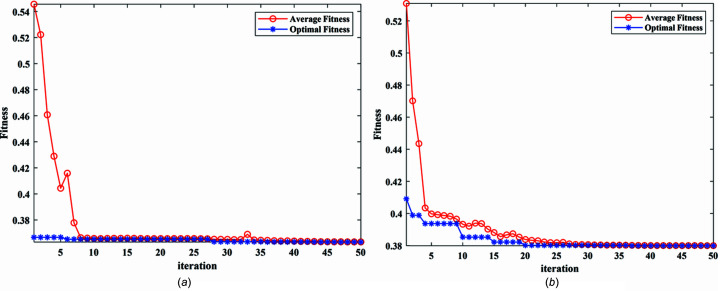
Optimization diagram of the genetic algorithm: (*a*) pitch; (*b*) roll.

**Figure 9:**
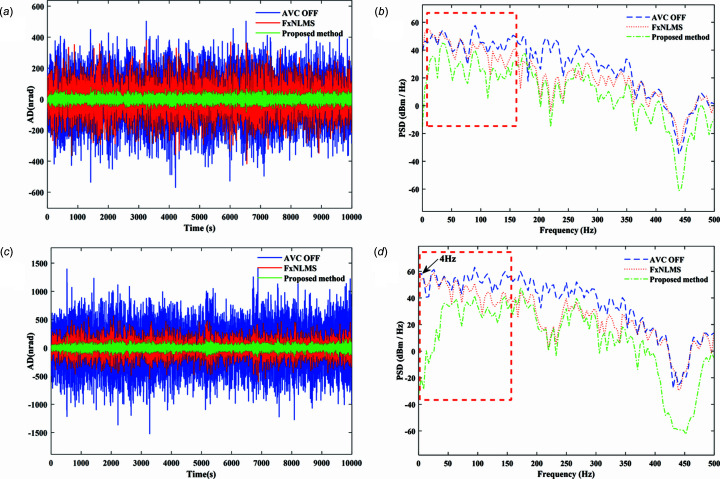
Vibration suppression performance comparison results at Bragg@16 keV: (*a*) time-domain results in the pitch direction; (*b*) frequency-domain (0–500 Hz) results in the pitch direction; (*c*) time-domain results in the roll direction; (*d*) frequency-domain (0–500 Hz) results in the roll direction.

**Figure 10:**
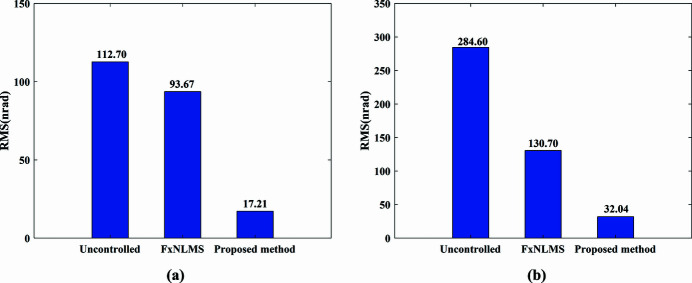
Vibration signal RMS results at Bragg@16 keV: (*a*) pitch direction; (*b*) roll direction.

The actual measured signal of the DCM at Bragg@16 keV operating mode is shown in Fig. 6 ▸. The measured vibration signal is decomposed into different frequency bands with the variable modal decomposition algorithm optimized by the GA, as shown in Fig. 7 ▸. An optimization diagram of the GA is shown in Fig. 8 ▸. Fig. 9 ▸ shows the vibration suppression performance of the FxNLMS algorithm and proposed method at Bragg@16 keV operating mode. Fig. 10 ▸ shows the vibration signal’s RMS values under the FxNLMS algorithm and proposed method at Bragg@16 keV.

From Figs. 9 ▸(*a*) and 10 ▸(*a*), it can be seen that the proposed adaptive vibration control method has a significant advantage over the FxNLMS algorithm in terms of vibration suppression in the pitch direction at Bragg@16 keV. The FxNLMS algorithm decreased the angular displacement in the pitch direction by 16.89%, while the proposed method reduced the angular displacement in the pitch direction by 84.73%. Similarly, from Figs. 9 ▸(*c*) and 10 ▸(*b*), the FxNLMS algorithm decreased the angular displacement in the roll direction by 54.08%, while the proposed method reduced the angular displacement in the roll direction by 88.74%. From Figs. 9 ▸(*b*) and 9 ▸(*d*), in the frequency range 0–150 Hz, the proposed method shows a ∼10 dB decrease in the pitch direction and ∼20 dB reduction in the roll direction. Consequently, the proposed method has a satisfactory vibration suppression performance at low frequencies. On the contrary, the FxNLMS algorithm has weak vibration damping ability at low frequencies.

### 3.2. Case 2

**Figure 11:**
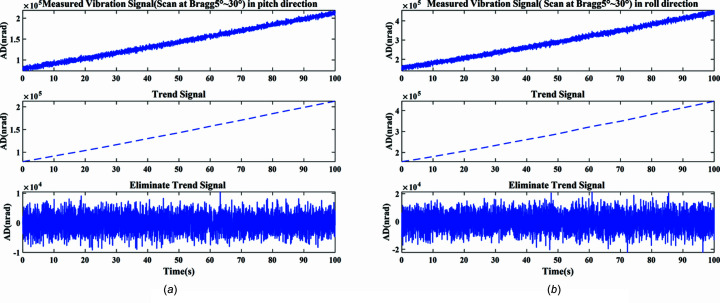
Actual measured signal of the DCM at 5–30° uniform scanning: (*a*) pitch direction; (*b*) roll direction.

**Figure 12:**
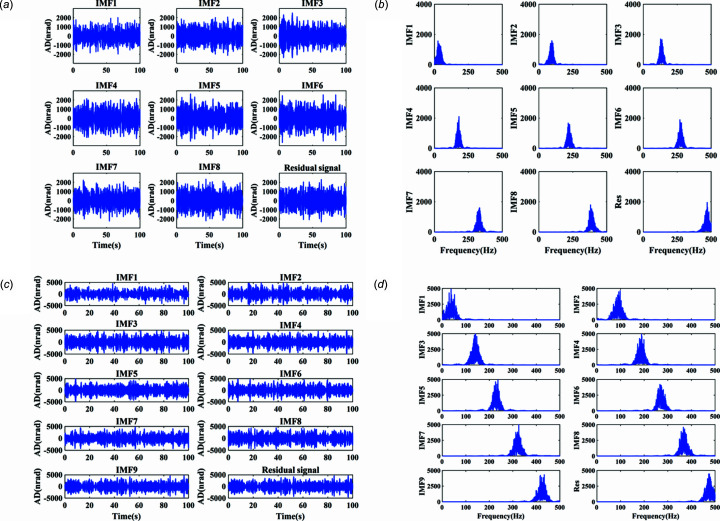
Decomposed signal and spectrum: (*a*) time-domain results in the pitch direction; (*b*) frequency-domain (0–500 Hz) results in the pitch direction; (*c*) time-domain results in the roll direction; (*d*) frequency-domain (0–500 Hz) results in the roll direction.

**Figure 13:**
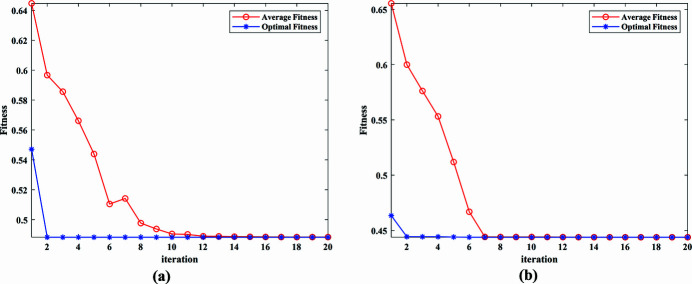
Optimization diagram of the genetic algorithm.

**Figure 14:**
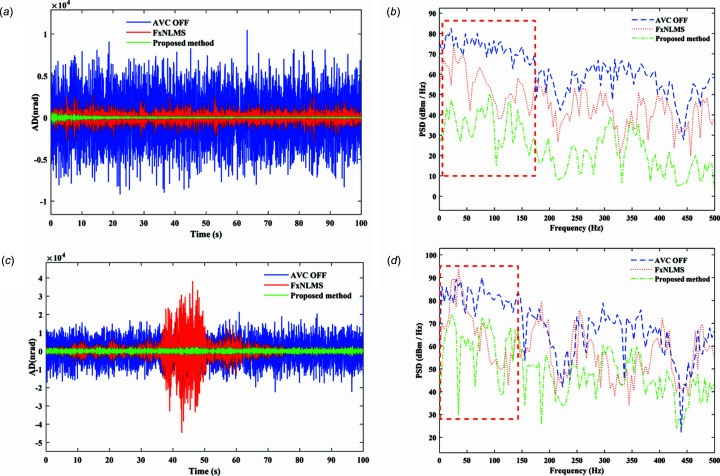
Vibration suppression performance comparison results at 5–30° uniform scanning: (*a*) time-domain results in the pitch direction; (*b*) frequency-domain (0–500 Hz) results in the pitch direction; (*c*) time-domain results in the roll direction; (*d*) frequency-domain (0–500 Hz) results in the roll direction.

**Figure 15:**
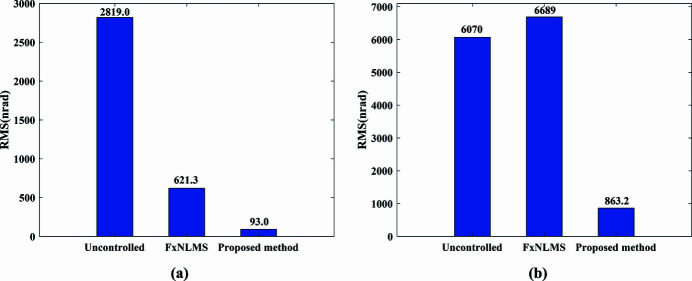
Vibration signal RMS value results at 5–30° uniform scanning: (*a*) pitch direction; (*b*) roll direction.

The actual measured signal of the DCM at 5–30° uniform scanning operating mode is shown in Fig. 11 ▸. The measured vibration signal is decomposed into different frequency bands with the variable modal decomposition algorithm optimized by GA, as shown in Fig. 12 ▸. The optimization diagram of the GA is shown in Fig. 13 ▸. Fig. 14 ▸ shows the vibration suppression performance of the FxNLMS algorithm and proposed method at 5–30° uniform scanning operating mode; Fig. 15 ▸ shows the vibration signal’s RMS values under the FxNLMS algorithm and proposed method at 5–30° uniform scanning.

It can be visualized from Figs. 14 ▸(*a*) and 15 ▸(*a*) that both the FxNLMS algorithm and the proposed method have vibration suppression effects. Compared with the FxNLMS algorithm, the proposed adaptive vibration control method decreases the angular displacement in the roll direction by 85.03%. From Figs. 14 ▸(*b*) and 14 ▸(*d*), in the frequency range 0–150 Hz, the proposed method shows ∼40 dB decrease in the pitch direction and ∼10 dB reduction shown in the roll direction. Consequently, the proposed method has a satisfactory vibration suppression performance at low frequencies. On the contrary, the FxNLMS algorithm has weak vibration damping ability at low frequencies. In particular, it can been seen from Fig. 14 ▸(*c*) that the FxNLMS algorithm suffers from control failure and vibration amplification in the pitch direction in the time range 35–50 s. Therefore, it can be seen that the stability and adaptive capability of the proposed adaptive control method has been relatively prominent.

## 4. Conclusion

This paper presents a novel method for adaptive vibration control based on VMD and the FxNLMS algorithm for DCMs at synchrotron radiation facilities. The VMD is optimized by a GA, and then a separate controller is designed for each decomposition signal based on the FxNLMS algorithm. The results show that the proposed adaptive vibration control method is superior to the conventional FxNLMS algorithm in terms of vibration suppression and convergence rate. Moreover, the process has a significant computational weight, which can be considered in the future to optimize the filter structure. This work should be of great significance for solving the stability of DCMs in the future. In future work, the application of the proposed method in practical beamline engineering will be promoted.

## Figures and Tables

**Table 1:** Computational expressions of the FxNLMS algorithm

Interference signal	*x*(*n*)
Primary path	*P*(*z*)
Primary path response	*d*(*n*) = *p* ^*T*^(*n*)*x*(*n*)
Secondary path	*S*(*z*)
Estimation of secondary path	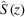
Finite impulse response filter	*W*(*z*)
Filter output signal	*y*(*n*) = *w* ^*T*^ *x*(*n*)
Anti-vibration signal	*y*′(*n*) = *s* ^*T*^(*n*)*y*(*n*)
Secondary path filtering signal	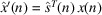
Residual signal update function (Madisetti, 2009 ▸)	*e*(*n*) = *d*(*n*) + *y*′(*n*) 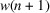 = 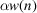 + 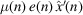
Adaptive learning rate	μ(*n*)

**Table 2:** Measurement sensor parameters

Brand	Germany / attocube
Model	IDS3010
Probe type	IDSHI1010632
Resolution	1 nm
Repeatability (vacuum state)	2 nm
Bandwidth	10 MHz

## References

[bb1] Akhtar, M. T., Abe, M. & Kawamata, M. (2004). *2004 47th Midwest Symposium on Circuits and Systems (MWSCAS’04)*, 25–28 July 2004, Hiroshima, Japan.

[bb3] Baker, R., Barrett, R., Clavel, C., Dabin, Y., Eybert-Berard, L., Mairs, T., Marion, P., Mattenet, M., Zhang, L., Baboulin, D. & Guillemin, J. (2013). *J. Phys. Conf. Ser.* **425**, 052015.

[bb2] Bao, W., Tu, X. & Li, F. (2020). *J. Vib. Meas. Diagn.* **40**, 272–277.

[bb4] Bian, J. (2017). *J. Propul. Technol.* **38**, 1618–1624.

[bb5] Dey, P., Satija, U. & Ramkumar, B. (2015). *2015 Annual IEEE India Conference (INDICON)*, 17–20 December 2015, New Delhi, India, pp. 1–5.

[bb6] Dragomiretskiy, K. & Zosso, D. (2014). *IEEE Trans. Signal Process.* **62**, 531–544.

[bb7] Fallah, M. & Moetakef-Imani, B. (2019). *Mech. Syst. Signal Process.* **129**, 91–111.

[bb11] Jin, L., Wang, N., Zhu, W., Bian, F. & Xu, Z. (2017). *Nucl. Sci. Tech.* **28**, 159.

[bb12] Krishnan, S. R. & Seelamantula, C. S. (2013). *IEEE Trans. Signal Process.* **61**, 380–391.

[bb13] Li, J., Chen, Y., Zi, Y. & Pan, J. (2017). *Mech. Syst. Signal Process.* **85**, 512–529.

[bb15] Madisetti, V. K. (2009). *The Digital Signal Processing Handbook: Digital Signal Processing Fundamentals*, 2nd ed. New York: CRC Press.

[bb16] Madisetti, V. K. & Williams, D. (1999). *The Digital Signal Processing Handbook.* Boca Raton: CRC Press.

[bb17] Nocedal, J. & Wright, S. J. (2006). *Numerical Optimization*, 2nd ed. Berlin: Springer.

[bb18] Ram, R. & Mohanty, M. N. (2017). *Intl J. Nat. Comput. Res.* **6**, 17–35.

[bb19] Schafer, R. W. (2011). *IEEE Signal Process. Mag.* **28**, 111–117.

[bb20] Singh, A. & Harshit, M. (2014). *IOSR J. Comput. Eng.* **16**, 14–19.

[bb21] Sun, H. L., Zhang, P. Q., Gong, X. L. & Chen, H. B. (2007). *J. Sound Vibrat.* **300**, 117–125.

[bb23] Wang, F., Liu, C., Zhang, T., Dun, B., Han, Q. & Li, H. (2018). *J. Vib. Meas. Diagn.* **38**, 540–547.

[bb24] Wang, R., Markert, R., Xiang, J. & Zheng, W. (2015). *Mech. Syst. Signal Process.* **60–61**, 243–251.

[bb9] Wu, J., Gong, X., Song, Y., Chen, J., Zhu, W., Fan, Y., Qin, H. & Jin, L. (2021*a*). *Nucl. Instrum. Methods Phys. Res. A*, **985**, 164654.

[bb10] Wu, J., Gong, X., Song, Y., Chen, J., Zhu, W., Liu, Y., Fan, Y. & Jin, L. (2021*b*). *Nucl. Instrum. Methods Phys. Res. A*, **988**, 164872.

[bb8] Wu, Z. & Huang, N. E. (2009). *Adv. Adapt. Data Anal.* **01**, 1–41.

[bb25] Yamazaki, H., Ohashi, H., Senba, Y., Takeuchi, T., Shimizu, Y., Tanaka, M., Matsuzaki, Y., Kishimoto, H., Miura, T., Terada, Y., Suzuki, M., Tajiri, H., Goto, S., Yamamoto, M., Takata, M. & Ishikawa, T. (2013). *J. Phys. Conf. Ser.* **425**, 052001.

[bb26] Yi, S., Yang, B. & Meng, G. (2019). *Mech. Syst. Signal Process.* **114**, 644–657.

